# Dissecting Mechanochemistry III

**DOI:** 10.3762/bjoc.18.150

**Published:** 2022-10-12

**Authors:** Lars Borchardt, José G Hernández

**Affiliations:** 1 Department of Inorganic Chemistry, Ruhr-Universität Bochum, Universitätsstrasse 150, 44801 Bochum, Germany; 2 Grupo Ciencia de los Materiales, Instituto de Química, Facultad de Ciencias Exactas y Naturales, Universidad de Antioquia, Calle 70 No 52-21, Medellín, Colombia

In the past ten years, the use of mechanochemical techniques (e.g., grinding, milling, extrusion, pulsed ultrasonication, resonant acoustic mixing, etc.) have widespread in the field of organic chemistry, enabling the development of new and more sustainable protocols for chemical synthesis [1–2]. Within this period, the *Beilstein Journal of Organic Chemistry* has organized two Thematic Issues (i.e., Mechanochemistry and Mechanochemistry II) to facilitate the open dissemination of the best research in the field of synthetic organic mechanochemistry. The great success of these past initiatives encouraged us to organize Mechanochemistry III, a new Thematic Issue in which the readership of the journal will find a collection of full research papers, letters and, for the first time, a Perspective article.

In more detail, the readers will find new mechanochemical protocols for the halogenation of organic substrates. For example, Banerjee and co-workers reported the mono-, di-, and trihalogenation of aromatics by controlling the stoichiometry of the *N*-halosuccinimide (NXS) and PEG-400 as the grinding auxiliary in a mechanical grinder (Scheme 1a) [3].

**Scheme 1:**
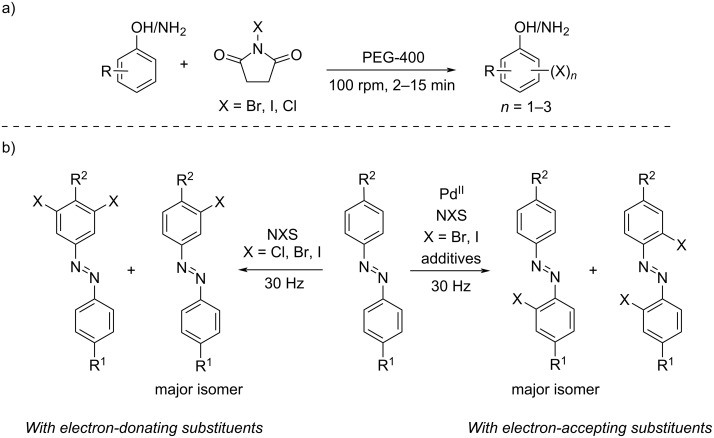
a) Mechanochemical PEG-400-assisted halogenation of phenols and anilines using NXS. b) Halogenation of azobenzenes with NXS.

*N*-Halosuccinimides were also key reagents to develop the mechanochemical halogenation of azobenzenes as studied by Ćurić and co-workers [4]. They demonstrated how, depending on the azobenzene structure, the halogenation of the C–H bonds with NBX occurred in the presence of Pd(II) catalysts or under metal-free conditions (Scheme 1b). Similarly, in the absence of metal catalysts, *N*-fluorobenzenesulfonimide (NFSI) was found to act as a mild fluorinating reagent for activated aromatics by mechanochemistry [5]. Such a collective effort to access halogenated substrates is understandable, owning the synthetic value of organic halides as substrates in multiple reactions. For instance, within this Thematic Issue, the synthetic relevance of aryl halides was evidenced during the development of a protocol for the solid-state palladium-catalyzed borylation reported by Kubota, Ito, and co-workers (Scheme 2) [6].

**Scheme 2:**
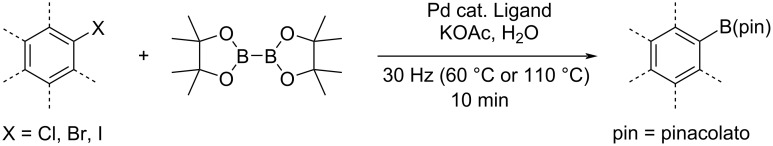
Mechanochemical palladium-catalyzed borylation protocol of aryl halides.

Moreover, Štrbac and Margetić used dibrominated polycyclic imides as substrates to generate reactive alkenes, which could be trapped in situ by several dienes through Diels−Alder cycloadditions by ball milling (Scheme 3) [7].

**Scheme 3:**
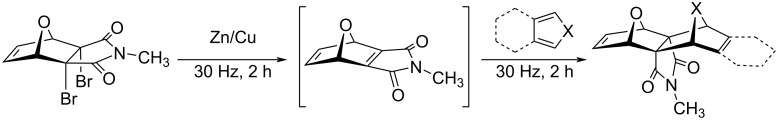
1,2-Debromination of polycyclic imides, followed by in situ trapping of the dienophile by several dienes.

Further, Moores and co-workers synthesized phosphorus-bridged heptazine-based carbon nitrides (g-h-PCN) from an initial mechanical treatment of trichloroheptazine and Na_3_P, once again highlighting the importance of halogenated organic molecules as building blocks for graphitic heptazine materials (Scheme 4) [8].

**Scheme 4:**
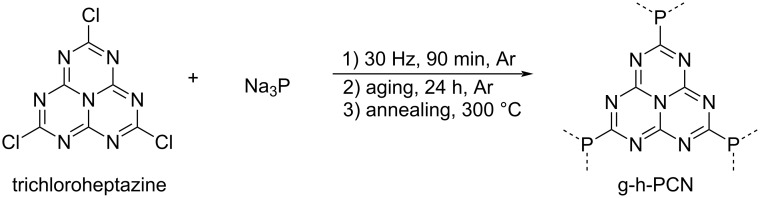
Synthesis of g-h-PCN from sodium phosphide and trichloroheptazine mediated by mechanochemistry.

Another halogenated molecule, 2,3-dichloro-5,6-dicyano-1,4-benzoquinone (DDQ), proved to be an appropriate oxidant for C–N couplings towards the synthesis of 1,2-disubstituted benzimidazoles and quinazolin-4(3*H*)-one derivatives under mechanochemical conditions, as evidenced by Mal and co-workers (Scheme 5) [9].

**Scheme 5:**
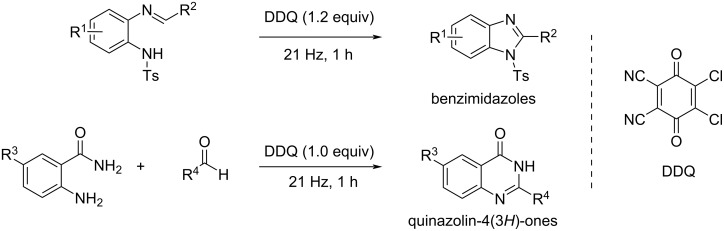
Mechanochemical intra- and intermolecular C–N coupling reactions using DDQ as an oxidant.

However, the findings within Mechanochemistry III span beyond the synthesis or the use of halogenated compounds and include the mechanochemical preparation of isocyanides [10], formylated and acetylated amines [11], and the mechanosynthesis of unsymmetrical salens ligands for preparing metal–salen catalysts [12]. This illustrates the broad applicability of mechanochemical activation to advance chemical synthesis on various fronts. Therefore, we hope you enjoy reading the variety of manuscripts in this Thematic Issues as much as we enjoyed editing it, which was only possible thanks to the impeccable assistance by the Editorial Team of the Beilstein-Institut.

Lars Borchardt and José G. Hernández

Bochum, Medellín, October 2022

## ORCID® iDs

Lars Borchardt - https://orcid.org/0000-0002-8778-7816

José G. Hernández - https://orcid.org/0000-0001-9064-4456

## References

[1] Friščić T, Mottillo C, Titi H M (2020). Angew Chem, Int Ed.

[2] Ardila-Fierro K J, Hernández J G (2021). ChemSusChem.

[3] Das D, Bhosle A A, Chatterjee A, Banerjee M (2022). Beilstein J Org Chem.

[4] Barišić D, Pajić M, Halasz I, Babić D, Ćurić M (2022). Beilstein J Org Chem.

[5] Hernández J G, Ardila-Fierro K J, Barišić D, Geneste H (2022). Beilstein J Org Chem.

[6] Kubota K, Baba E, Seo T, Ishiyama T, Ito H (2022). Beilstein J Org Chem.

[7] Štrbac P, Margetić D (2022). Beilstein J Org Chem.

[8] Fiss B G, Douglas G, Ferguson M, Becerra J, Valdez J, Do T-O, Friščić T, Moores A (2022). Beilstein J Org Chem.

[9] Bera S K, Bhanja R, Mal P (2022). Beilstein J Org Chem.

[10] Basoccu F, Cuccu F, Casti F, Mocci R, Fattuoni C, Porcheddu A (2022). Beilstein J Org Chem.

[11] Casti F, Mocci R, Porcheddu A (2022). Beilstein J Org Chem.

[12] Zuo S, Zheng S, Liu J, Zou A Beilstein J Org Chem.

